# Protective activity of galacto-oligosaccharides against intestinal damage and inflammation induced by enterotoxigenic *Escherichia coli* F4^+^ and evaluation of prebiotic potential

**DOI:** 10.3389/fvets.2025.1740099

**Published:** 2026-02-02

**Authors:** Barbara Guantario, Sofie Tanghe, Alberto Finamore, Bert Devriendt, Chiara Devirgiliis, Stefanie Verstringe, Enya Rooyackers, Maartje De Vos, Jan Vande Ginste, Marianna Roselli

**Affiliations:** 1 Research Centre for Food and Nutrition, Consiglio per la ricerca in agricoltura e l'analisi dell'economia agraria (CREA), Rome, Italy; 2 Agrifirm, Drongen, Belgium; 3 Laboratory of Immunology, Faculty of Veterinary Medicine, Ghent University, Merelbeke, Belgium

**Keywords:** Caco-2 cells, intestinal permeability, NF-κB activation, pathogen infection, tight junction proteins

## Abstract

Post-weaning diarrhea in piglets is frequently caused by enterotoxigenic *Escherichia coli* (ETEC) F4^+^. The objective of this study was to examine the possible protective effect of galacto-oligosaccharides (GOS) on ETEC F4^+^-induced intestinal injury. Growth inhibition of ETEC F4^+^ in the presence of 2% GOS was assessed, as well as the ability of GOS to reduce pathogen adhesion and invasion in the intestinal Caco-2 cell line. GOS ability to counteract ETEC F4^+^ adhesion was also assessed *ex vivo* in piglet small intestinal villi. Protective activity of GOS against ETEC F4^+^-induced membrane damage in Caco-2 cells was evaluated through transepithelial electrical resistance (TEER), phenol red apparent permeability (Papp) and immunolocalization of tight junction proteins occludin and ZO-1. Inflammation was assessed by quantification and immunolocalization of phosphorylated-p65 protein, indicative of NF-κB activation. Finally, GOS prebiotic activity on probiotic strains *Lactobacillus amylovorus*: ATCC 33198 and DSM 16698, as well as *Limosilactobacillus reuteri* subsp. *porcinus* DSM 110571, was also investigated. The results showed that GOS significantly reduced ETEC F4^+^ adhesion and invasion in Caco-2 cells, as well as adhesion to piglet intestinal villi. Furthermore, GOS markedly decreased ETEC F4^+^ induced membrane damage, as evidenced by improvement of TEER, phenol red Papp and tight junction protein immunolocalization. A reduction of p65 phosphorylation and nuclear translocation in the presence of GOS indicated a diminished activation of the NF-κB pathway. GOS also exhibited promising prebiotic activity toward the tested probiotic strains. Taken together, these *in vitro* findings suggest the potential of including GOS in piglet weaner diets to prevent ETEC F4^+^-induced intestinal injury.

## 1 Introduction

Weaning is widely acknowledged as a critical stage in the piglets life, exposing them to multiple stressors, which can have cumulative effects on their health and physiological development. The separation of piglets from the sow introduces abrupt dietary and environmental changes that impact gut microbiota and immune system maturation, as well as compromise intestinal barrier integrity, leading to dysbiosis and increased susceptibility to gut disorders, gastrointestinal disturbances, infections and diarrhea (1). In particular, post-weaning diarrhea poses a critical economic threat to the global swine industry, causing morbidity, mortality, and substantial production losses (2, 3).

Enterotoxigenic *Escherichia coli* (ETEC) F4^+^ represents one of the most common etiologic agents of post-weaning diarrhea, accounting for the high mortality rates occurring annually (4). To colonize the gut, ETEC F4^+^ adheres to host-specific receptors present on the enterocytes of the small intestine through its F4 fimbriae, or adhesins. Three serological variants of F4 have been described so far, namely F4ab, F4ac and F4ad, with the F4ac variant being the most prevalent in piglets (5). Once the pathogen is attached and has colonized the small intestine, it produces enterotoxins that lead to excessive secretion of water and electrolytes, ultimately resulting in watery diarrhea (6, 7).

Traditionally, antimicrobial growth promoters and high-dose zinc oxide were incorporated into weaner diets to mitigate post-weaning diarrhea and support growth, but the EU fully banned in-feed antibiotics in 2006 and restricted medicinal ZnO use in 2022, due to environmental concerns and the urgent need to halt antimicrobial resistance spreading (8, 9). Consequently, research into sustainable nutritional interventions and other preventive measures to reduce post-weaning diarrhea in piglets has intensified (10). Several feed-related measures have been widely investigated and applied, resulting in different degrees of efficacy, as reviewed by Canibe et al. (11). One of these strategies relies on the use of prebiotics, defined as “substrates selectively utilized by host microorganisms conferring a health benefit” (12). Among these substrates, galacto-oligosaccharides (GOS) are non-digestible carbohydrates, consisting of a complex mix of distinct galactose-containing oligosaccharides, which differ in terms of chain length (degree of polymerization) and type of glycosidic linkages between galactose or galactose-glucose moieties (13). GOS reach the colon intact, where they are fermented to produce short chain fatty acids (SCFA), that exert several health-promoting activities (14). In addition, GOS can shape gut microbiota composition by selectively promoting the growth of beneficial bacteria belonging to the former *Lactobacillus* and the *Bifidobacterium* genera (15). Thanks to this microbiota-dependent mode of action, GOS can indirectly prevent pathogen infections, as commensal bacteria can inhibit or limit the growth of harmful bacteria via competitive exclusion and/or nutrient availability. In addition, GOS can act through various microbiota-independent mechanisms: first, many of them structurally resemble the oligosaccharide residues of receptors on intestinal epithelial cells, to which pathogens adhere through their fimbriae. For instance, F4 fimbriae have been shown to bind galactose-containing glycosphingolipids of porcine small intestinal epithelium (16). As such, they might act as decoy receptors or antiadhesive molecules that competitively inhibit pathogen adherence and subsequent infection (17). Another relevant microbiota-independent pathway of GOS is through direct interaction with cell surface receptors present in epithelial or innate immune cells in the intestinal mucosa, activating cell signaling that reinforces the epithelial barrier, as reviewed by Mavrogeni et al. (18). Finally, another possible intriguing feature of GOS relies on their immunomodulatory properties (19, 20). In summary, GOS can be considered a promising and sustainable alternative to antibiotics in weaned piglets. By fostering a healthier microbial environment and strengthening the gut lining, GOS can mitigate the weaning adverse effects and improve resilience against post-weaning diarrhea, as well as support overall gastrointestinal development (21, 22).

Based on the above mentioned information, we hypothesized that GOS, next to their prebiotic function, could have a direct microbiota-independent protective effect against ETEC F4^+^ infection in an *in vitro* intestinal cell model. Hence, the aim of the present study was to evaluate whether GOS could prevent ETEC F4^+^ adhesion and invasion, as well as ETEC F4^+^-induced intestinal barrier injury and NF-κB translocation in the epithelial Caco-2 cell line. These cells are able to differentiate *in vitro* and achieve the morpho-functional characteristics of mature enterocytes, with brush border microvilli and epithelial cellular junctions (23). Additionally, pathogen adhesion was assessed in piglet intestinal villi, an *ex vivo* model better reproducing the gut mucosal environment. Finally, GOS potential prebiotic activity toward three different probiotic *Lactobacillus* and *Limosilactobacillus* strains of pig origin was investigated.

## 2 Materials and methods

### 2.1 Intestinal Caco-2 cell culture conditions

Caco-2 cells, obtained from INSERM (Paris, France), were subcultured at low density and used between passage 90 and 105, according to Natoli et al. (24). Cells were maintained at 37 °C in a 95% air/5% CO_2_ atmosphere at 90% relative humidity in Dulbecco's modified Eagle's medium (DMEM) containing 25 mM glucose, 3.7 g/l NaHCO_3_, 4 mM L-glutamine, 1% non-essential amino acids, 1 × 10^5^ U/l penicillin, 100 mg/l streptomycin, 10% heat inactivated fetal bovine serum (FBS, Euroclone, Milan, Italy). Cell culture media and reagents were purchased from Corning (Milan, Italy), unless otherwise stated. For experiments, cells were seeded in 24-well plates (Becton Dickinson, Milan, Italy) or polyethylene terephthalate permeable filters (Falcon™ 10.5 mm diameter, 0.4 μm pore size, Becton Dickinson, Milan, Italy), which allow separation between apical (AP) and basolateral (BL) compartments. After confluency, cells were left for 17–21 days to allow complete differentiation. Medium was changed 3 times a week.

### 2.2 GOS preparation

**Figure 1:**
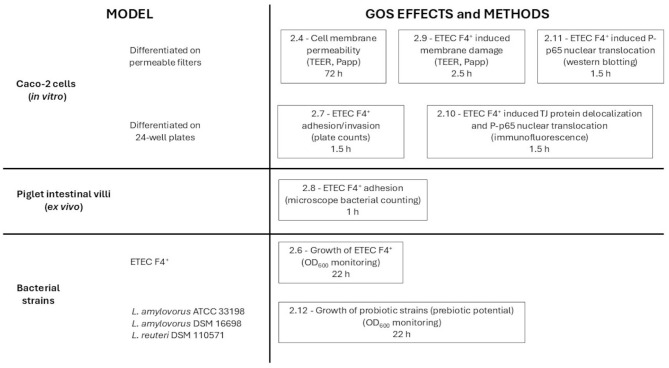
Schematic representation of the experimental design. The models and the methods used are indicated, with references to the corresponding paragraph numbers of the Materials and Methods section. ETEC F4^+^, enterotoxigenic *Escherichia coli* F4^+^; *L. amylovorus* ATCC 33198, *Lactobacillus amylovorus* ATCC 33198; *L. amylovorus* DSM 16698, *Lactobacillus amylovorus* DSM 16698; *L. reuteri* DSM 110571, *Limosilactobacillus reuteri* subsp. *porcinus* DSM 110571; Papp, apparent permeability; P-p65, phosphorylated p65 protein; TEER, transepithelial electrical resistance; TJ, tight junction.

The GOS product, synthesized by β-galactosidase through the lactose transgalactosylation reaction, was provided as a powder by Royal Agrifirm Group (START+; Royal Agrifirm Group, Drongen, Belgium). For all experiments, GOS powder was weighed, resuspended in experimental medium (bacterial or cell growth medium) at 2% final concentration, and filtered with 0.45 μm syringe filters (Merck Millipore, Darmstadt, Germany). The experimental design is outlined in Figure 1.

### 2.3 Cell membrane permeability measurements

Caco-2 cells were differentiated on permeable filters (1 × 10^6^ cells/filter), as described above. Cell membrane permeability was assayed by measuring the transepithelial electrical resistance (TEER), according to Srinivasan et al. (25), with a Millicell Electrical Resistance system (Merck Millipore, Darmstadt, Germany). TEER values were checked before each experimental assay, to verify correct TJ formation and complete cell differentiation into mature enterocytes. TEER measurements were carried out at different time points, depending on the experiments, as detailed below. For each time point, the TEER values of treated samples were expressed as % of the TEER values of control (untreated) cells, set at 100%. Cell permeability was also measured at the end of treatments by measuring the phenol red passage, as reported by Monastra et al. (26). Briefly, after three washes with phosphate buffered saline containing Ca^++^ and Mg^++^ (PBS^++^), 0.5 ml 1 mM phenol red was added to the AP compartment of cell monolayers, whereas 1 ml PBS^++^ was added in the BL compartment. After 1 h incubation at 37 °C, 0.9 ml BL medium was collected, added with 0.1 ml 0.1 N NaOH, and read at 560 nm (Infinite M200 microplate reader, Tecan, Milan, Italy) to determine the phenol red concentration passed from AP to BL compartment. This concentration was used to calculate the phenol red apparent permeability coefficient (Papp) by applying the following formula: Papp = *Ct* × *V*_BL_/Δ*t* × ·*C*0 × *A*, where *V*_BL_ is the volume of the BL compartment (cm^3^), *A* is the filter area (cm^2^), Δ*t* is the time interval (s), *Ct* is the phenol red concentration in the BL compartment at the end of time interval, and *C*0 is the phenol red concentration in the AP compartment at the beginning. The TJ were considered open and indicative of an absence of cell monolayer integrity when the phenol red Papp values were above 1 × 10^−6^ cm s^−1^, as determined by previous reports in the literature (27). Differences observed among samples below this threshold value were thus considered biologically irrelevant, irrespective of statistical significance.

### 2.4 GOS toxicity assay on Caco-2 cells

In order to assess the potential toxicity of GOS, Caco-2 cells, differentiated on permeable filters, were apically treated with 2% GOS. TEER was recorded for up to 72 h, and phenol red Papp was assayed at the end of treatment. Two independent experiments, carried out in triplicate, were performed.

### 2.5 Bacterial strains and growth conditions

The intestinal pathogen F4^+^ enterotoxigenic *Escherichia coli* strain (ETEC F4^+^, O149:F4ac^+^, LT^+^, STa^+^, STb^+^), provided by the Lombardy and Emilia Romagna Experimental Zootechnic Institute (Reggio Emilia, Italy) was grown in Luria-Bertani (LB) broth at 37 °C. The ETEC F4^+^ strain GIS26 (O149:F4ac^+^, LT^+^, STa^+^, STb^+^) was grown on brain heart infusion (BHI) agar plates for 16 h at 37 °C.

*Lactobacillus amylovorus* ATCC 33198 (LMG9434), *Lactobacillus amylovorus* DSM 16698 and *Limosilactobacillus reuteri* subsp. *porcinus* DSM 110571 were grown in De Man Rogosa Sharpe (MRS) medium (supplemented with 0.5 g/l L-cysteine for DSM 110571), for 24 h at 37 °C under microaerophilic conditions.

All media and supplements were provided by Oxoid (Thermo Fisher, Waltham, MA, USA), unless otherwise stated.

### 2.6 ETEC F4^+^ growth in media containing GOS

A liquid broth assay was performed to evaluate if ETEC F4^+^ growth could be affected by the presence of GOS in LB or antibiotic- and FBS-free DMEM media. Overnight cultures of ETEC F4^+^ were diluted 1:50 in the two media containing or not 2% GOS and then dispensed into 96-well plates at a final volume of 200 μl per well. Bacterial growth was monitored by recording the OD_600_ of the bacterial cultures for 22 h at 1 h intervals using an automated plate reader (Infinite M200, Tecan, Milan, Italy). The OD_600_ values were normalized with respect to the medium alone. One independent experiment was conducted in triplicate.

### 2.7 ETEC F4^+^ adhesion and invasion assays on Caco-2 cells

The adhesion and invasion assays were carried out on Caco-2 cells, differentiated in 24-well plates (1 × 10^6^ cells/well). Cells were placed in antibiotic- and FBS-free DMEM 16 h before the experiment. On the day of the assay, overnight bacterial cultures of ETEC F4^+^ were diluted 1:20 in LB broth and grown for approximately 3 h up to the exponential growth phase, as monitored by OD_600_ reading. Appropriate amounts of bacterial cells were harvested by centrifugation at 5,000 rpm for 10 min, resuspended in antibiotic- and FBS-free cell culture medium, pre-incubated with GOS or DMEM for 10 min at 37 °C and then added to cell monolayers at a concentration of 1 × 10^8^ colony forming units (CFU)/well (approximately 100:1 bacteria to cell ratio). Co-cultures of bacteria and Caco-2 cells were incubated at 37 °C for 1.5 h. In the adhesion assay, non-adhering bacteria were removed by 5 washes with Hanks' Balanced Salt solution (HBSS: 137 mM NaCl, 5.36 mM KCl, 1.67 mM CaCl_2_, 1 mM MgCl_2_, 1.03 mM MgSO_4_, 0.44 mM KH_2_PO_4_, 0.34 mM Na_2_HPO_4_, 5.6 mM glucose) and cell monolayers were lysed with 1% Triton-X-100, according to Roselli et al. (28).

In the invasion assay, after the 1.5 h infection period, co-cultures of bacteria and Caco-2 cells were washed twice with HBSS and then incubated with antibiotic- and FBS-free DMEM containing gentamicin sulfate (50 mg/L; Merck, Darmstadt, Germany) for additional 2.5 h, to kill residual viable extracellular bacteria. After gentamicin incubation, Caco-2 cells were washed and lysed as described above. For both adhesion and invasion assays, viable bacterial cells (adhered and invading, respectively) were quantified by plating appropriate serial dilutions of Caco-2 lysates on LB agar. CFU were counted on plates after overnight incubation at 37 °C.

For both assays, at least two independent experiments were performed in duplicate.

### 2.8 *Ex vivo* villus adhesion inhibition assay

The villus adhesion assay was performed as described by Rasschaert et al. (29). Small intestinal villi were collected from the mid-jejunum of a healthy piglet (6 weeks old female, Belgian Landrace x Piétrain, weaned at 4 weeks), euthanized for an unrelated experimental trial, approved by the Ethical Committee of the Faculties of Veterinary Medicine and Bioscience Engineering at Ghent University (approval number EC2019/085). No additional animals were used or sacrificed for this study. The piglet was genotyped for the F4 receptor presence using the MUC13 SNP assay (30). Small intestinal villi were incubated with 4 × 10^8^ CFU of the ETEC F4^+^ strain GIS26 for 1 h at room temperature. To assess the ability of GOS to inhibit adhesion of ETEC F4^+^ to the small intestinal epithelium, the bacteria were pre-incubated with 0.5 mg/ml GOS for 1 h prior to performing the adhesion assay. As a positive control for inhibition of adhesion, the bacteria were incubated with a FaeGac-specific monoclonal antibody (mAb in house, clone IMM01). The adhesion of the bacteria to the villus epithelium was then evaluated by counting the number of bacteria adhering along a 50 μm villous brush border length at 5 randomly selected places, with a phase-contrast microscope at a 600 × magnification. The number of bacteria per 250 μm villous brush border length was then calculated. This procedure was performed three times, in a single experiment. The % inhibition was calculated by dividing the number of adherent bacteria in the presence of GOS or mAb by the number of adherent bacteria in the absence of GOS.

### 2.9 Evaluation of effects of ETEC F4^+^ and GOS on Caco-2 TEER and phenol red Papp

Caco-2 cells differentiated on permeable filters (1 × 10^6^ cells/filter) were apically treated with ETEC F4^+^ (1 × 10^8^ CFU/well), prepared as described above. Appropriate amounts of bacterial cells were centrifuged, resuspended in antibiotic- and FBS-free cell culture medium, pre-incubated with GOS or DMEM for 10 min at 37 °C and then apically added to cell monolayers. TEER was recorded every 30 min for up to 2.5 h, and phenol red Papp was assayed at the end of treatments. Three independent experiments, carried out at least in duplicate, were performed.

### 2.10 Immunolocalization of TJ (ZO-1 and Occludin) proteins and P-p65

To assess the effect of GOS on ETEC-induced TJ protein delocalization, Caco-2 cells, differentiated on glass coverslips in 24-well plates (1 × 10^6^ cells/well), were treated with 2% GOS in the presence or absence of ETEC F4^+^ (1 × 10^8^ CFU/well), prepared as described above, for 1.5 h at 37 °C. At the end of the experiment, Caco-2 cells were washed with cold PBS^++^, fixed in ice-cold methanol for 3 min, and then incubated with rabbit polyclonal anti-ZO-1 or mouse monoclonal anti-occludin (Zymed Laboratories, San Francisco, CA, USA) or rabbit polyclonal anti-P-p65 (Cell Signaling Technology, Danvers, MA, USA) for 1 h. For secondary detection, cells were incubated with tetramethylrhodamine isothiocyanate (TRITC) conjugated secondary antibodies (Jackson Immunoresearch, Milan, Italy), for 1 h. 4′,6-diamidino-2-phenylindole (DAPI) was used to label DNA in nuclei. Stained monolayers were mounted on glass slides using the Prolong Gold antifade reagent (Molecular Probes, Invitrogen, Milan, Italy) and analyzed using a confocal fluorescence microscope (LSM 700, Zeiss, Jena, Germany). Three independent assays were conducted.

### 2.11 Western blotting assay of p65 NF-κB subunit

To assess the effect of GOS on ETEC F4^+^-induced p65 phosphorylation, Caco-2 cells differentiated on permeable filters (1 × 10^6^ cells/filter) were apically treated with 2% GOS in the presence or absence of ETEC F4^+^ (1 × 10^8^ CFU/well), prepared as above described, for 1.5 h at 37 °C. At the end of treatments, cells were washed twice with cold PBS and lysed in cold radioimmunoprotein assay buffer (RIPA: 20 mM Tris-HCl pH 7.5, 150 mM NaCl, 0.1% SDS, 1% Na deoxycholate, 1% Triton X-100), supplemented with 1 mm phenylmethylsulphonyl fluoride, protease inhibitor (Complete Mini, Roche, Milan, Italy) and phosphatase inhibitor (PhosSTOP, Roche, Milan, Italy) cocktails. Cell lysates (50 μg total proteins, quantified by Lowry Assay) were dissolved in sample buffer (50 mM Tris-HCl pH 6.8, 2% SDS, 10% glycerol, 100 g/L bromophenol blue, 10 mm β-mercaptoethanol), heated for 5 min, fractionated by SDS-polyacrylamide gel (4–20% gradient) electrophoresis and transferred to nitrocellulose filters (Trans-Blot Turbo, Biorad, Milan, Italy). Membranes were incubated with the following primary antibodies: rabbit monoclonal anti-human NF-κB total p65 (clone D14E12) or P-p65 (Ser536, clone 93H1) or mouse monoclonal anti-human α-tubulin (clone DM1A), all from Cell Signaling Technology (Danvers, MA, USA). Proteins were detected with horseradish peroxidase-conjugated secondary antibodies (Cell Signaling Technology, Danvers, MA, USA) and enhanced chemiluminescence reagent (ECL kit Lite Ablot Extend, Euroclone, Milan, Italy), followed by the analysis of chemiluminescence with the charge-coupled device camera detection system Las4000 Image Quant (GE Health Care Europe GmbH, Milan, Italy). Relative levels of the phosphorylated p65 protein were normalized to their corresponding unphosphorylated form. To verify equal protein loading, the housekeeping protein α-tubulin was used as internal control (Supplementary Figure S1). Three independent experiments, carried out in triplicate, were performed.

### 2.12 Prebiotic activity of GOS

The experiments were conducted using the probiotic strains *Lactobacillus amylovorus* ATCC 33198 (LMG9434), *L. amylovorus* DSM 16698 and *Limosilactobacillus reuteri* subsp. *porcinus* DSM 110571. Each probiotic strain, pre-grown overnight in MRS at 37 °C, was inoculated at 1:500 dilution (*L. amylovorus* ATCC 33198 and DSM 16698) or diluted to OD = 1 and inoculated at 1% (v/v; *L. reuteri* subsp. *porcinus* DSM 110571) in MRS medium without glucose (MRSnoG, Liofilchem, Italy) or added with 2% (w/v) glucose (used as reference carbon source) or added with 2% (w/v) GOS (used as test carbon source), in separate tubes. The MRSnoG medium served as negative control. Each *inoculum* was dispensed into 96-well plates at a final volume of 200 μl (*L. amylovorus* ATCC 33198 and DSM 16698) or 300 μl (*L. reuteri* subsp. *porcinus* DSM 110571) per well, in triplicate. Bacterial growth was monitored by recording the OD_600_ of the cultures for 22 h at 1 h intervals using an automated plate reader (Infinite M200, Tecan, Milan, Italy; or Multiskan FC, Life Technologies, Merelbeke, Belgium). The OD_600_ values were normalized with respect to the MRS medium alone, representing the blank. One independent experiment was conducted in triplicate, for each strain.

### 2.13 Statistical analysis

The statistical significance of the differences was evaluated by one-way ANOVA followed by *post hoc* Tukey HSD test, after verifying normality and homogeneity of variance by Shapiro–Wilk's and Levene's tests, respectively. For ETEC F4^+^ growth curve and adhesion/invasion assays, Student's *t*-test was applied. In the figures, mean values with different superscript letters or asterisks (for ANOVA or *t*-test, respectively) significantly differ. Statistical significance was set at *P* values < 0.05. The statistical analyses were executed with Microsoft Office Excel 2011 upgraded with XLSTAT (version 4 March 2014).

## 3 Results

### 3.1 Maintenance of Caco-2 cell monolayer integrity following GOS treatment

GOS was preliminarily tested for its potential to directly impact on ETEC F4^+^ growth in LB and DMEM media (Supplementary Figure S2). In both media, the presence of 2% GOS enhanced pathogen growth during the exponential phase (2 h, 4 h and 8 h time points, *P* < 0.01 within each time point), while at late stationary phase (22 h time point), growth kinetics resulted to decrease in LB (Supplementary Figure S2A, *P* < 0.01), or be unchanged in DMEM (Supplementary Figure S2B), with respect to the corresponding media alone. We therefore concluded that GOS had no relevant direct inhibitory effect on ETEC F4^+^ growth.

**Figure 2:**
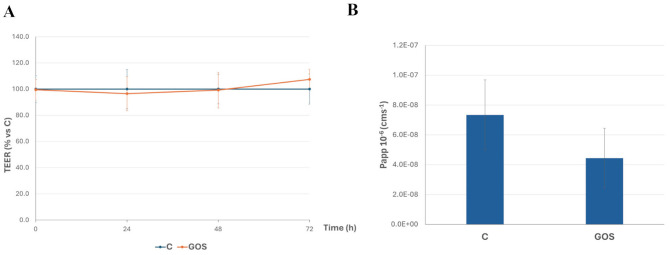
GOS effects on Caco-2 cells monolayer integrity: Transepithelial electrical resistance (TEER, **A**) and phenol red apparent permeability (Papp, **B**). Cells, differentiated on permeable filters, were untreated (Control, C) or treated with GOS (2%). TEER values, recorded every 24 h for up to 72 h, were calculated as % of the TEER value of control filters, for each time point. Phenol red Papp was measured at 72 h, and values are reported as cm s^−1^. Values represent means ± SD of two independent experiments, carried out in triplicate.

In order to evaluate if GOS exposure could perturb intestinal epithelial permeability, TEER and phenol red Papp were measured in differentiated Caco-2 cells after treatment with 2% GOS, apically added to cell monolayers for up to 72 h. The results show that no significant differences could be observed between treated and untreated (control) cells, in terms of TEER measurements (Figure 2A) and phenol red Papp (Figure 2B), indicating that monolayers integrity was not affected by 2% GOS treatment. Indeed, permeability coefficients lower than the 1 × 10^–6^ cm s^–1^ threshold are considered indicative of intact cell monolayers, irrespective of differences among samples that are below this threshold.

### 3.2 Effectiveness of GOS in reducing ETEC F4^+^ adhesion to and invasion of Caco-2 cells

**Figure 3:**
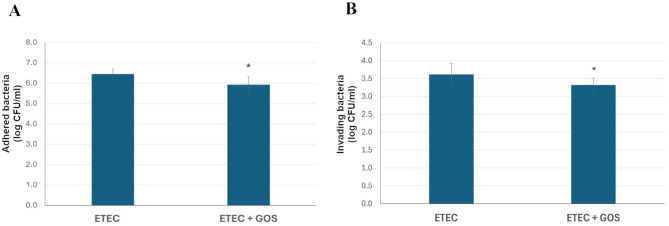
Reduction of enterotoxigenic *Escherichia coli* (ETEC) F4^+^ adhesion **(A)** and invasion **(B)** in differentiated Caco-2 cells mediated by GOS. ETEC F4^+^ was allowed to adhere or invade Caco-2 cells, either alone (ETEC) or in the presence of 2% GOS (ETEC + GOS). Data are reported as log colony forming units (CFU)/ml recovered after plating. Columns represent the mean ± SD of at least two independent experiments, each performed in technical duplicate. Statistical analysis was performed by Student's *t*-test (*P < 0.001 and *P* < 0.01 for adhesion and invasion assays, respectively).

The ETEC F4^+^ strain is known to be able to adhere to and being internalized by Caco-2 cells, with a number of recovered cells in the order of magnitude of 10^6^ CFU/ml and 10^3^ CFU/ml after adhesion and invasion, respectively, with initial bacterial loads of 1 × 10^8^ CFU/ml (28). The potential capacity of 2% GOS to counteract ETEC F4^+^ adhesion and invasion was investigated in Caco-2 cells. Figure 3A shows the results of the adhesion assay, indicating that the presence of 2% GOS significantly reduced pathogen adhesion, with a decrease of approximately 0.5 log CFU/ml, as compared to pathogen alone (*P* < 0.001). A similar reduction activity was also observed in the pathogen invasion assay, as shown in Figure 3B, where the number of invading ETEC F4^+^ cells was significantly reduced by approximately 0.5 log CFU/ml, as compared to pathogen alone (*P* < 0.01).

### 3.3 Reduction by GOS of ETEC F4^+^ adhesion to piglet small intestinal villi

**Figure 4:**
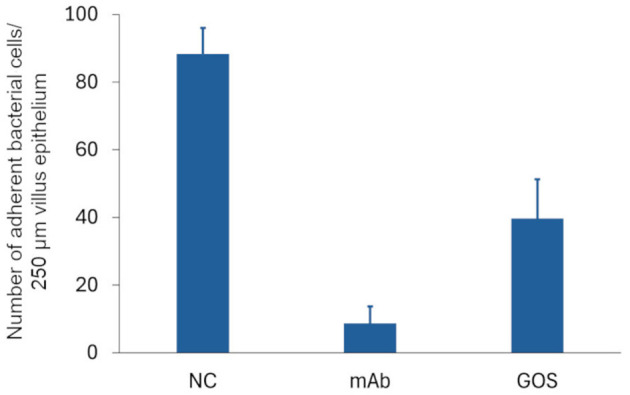
GOS reduces adhesion of ETEC F4^+^ to small intestinal villi. ETEC F4^+^ was incubated with medium (negative control, NC), a F4-specific monoclonal antibody (mAb) or GOS. The bacteria were then added to small intestinal villi. The number of bacteria adhering to the apical surface of the villi were counted. The data are presented as the mean of three technical replicates, the error bars represent the SD.

To evaluate the ability of GOS to inhibit the adhesion of ETEC F4^+^ to the porcine small intestinal epithelium, an *ex vivo* adhesion inhibition assay was performed. As shown in Figure 4, GOS reduced by 55% the amount of ETEC F4^+^ adhering to the villus epithelium. This suggests that GOS can inhibit the binding of ETEC F4^+^ to the small intestinal epithelium in pigs.

### 3.4 Protective effect of GOS against ETEC F4^+^-induced permeability increase and TJ protein delocalization in Caco-2 cells

**Figure 5:**
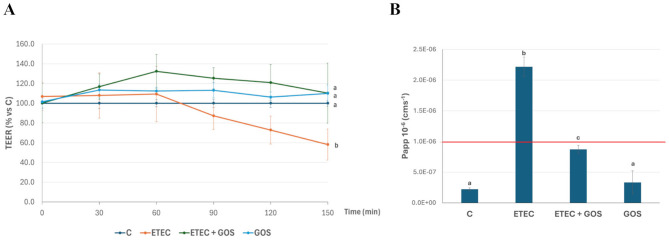
GOS effects on enterotoxigenic *Escherichia coli* (ETEC) F4^+^-induced permeability increase in Caco-2 cells: Transepithelial electrical resistance (TEER, **A**) and phenol red apparent permeability (Papp, **B**). Cells, differentiated on permeable filters, were untreated (Control, C), infected with ETEC F4^+^, or treated with 2% GOS, either alone or in combination with ETEC. TEER values were recorded every 30 min for up to 150 min. The TEER values of treated cells were expressed as % of the TEER value of control filters, for each time point. Phenol red Papp was measured at 150 min, and values are reported as cm s^−1^. A red line set at 1 × 10^−6^ cm s^−1^ represents Papp threshold, indicating destroyed cell monolayer integrity for values above. Values represent means ± SD of three independent experiments, carried out at least in duplicate. Statistical analysis was performed only at final time points (2.5 h). Means without a common letter significantly differ, *P* < 0.001.

ETEC F4^+^ is known to affect Caco-2 cell monolayer integrity, with a TEER decrease starting at 90 min after AP infection (28). The potential GOS ability to counteract the ETEC F4^+^-induced permeability increase was investigated in Caco-2 cells. As expected, ETEC F4^+^ infection induced a significant TEER decrease (Figure 5A, *P* < 0.05), as compared to untreated (control) and GOS-treated cells, either alone or in combination with pathogen infection (Figure 5A). This decrease was associated with a biologically relevant phenol red Papp increase, as the corresponding values were in the order of magnitude of 2.2 × 10^−6^ cm s^−1^, indicating that the TJ were open, while the cells co-incubated with ETEC F4^+^ and GOS showed phenol red Papp values comparable to untreated and GOS-treated cells (Figure 5B, *P* < 0.001).

**Figure 6:**
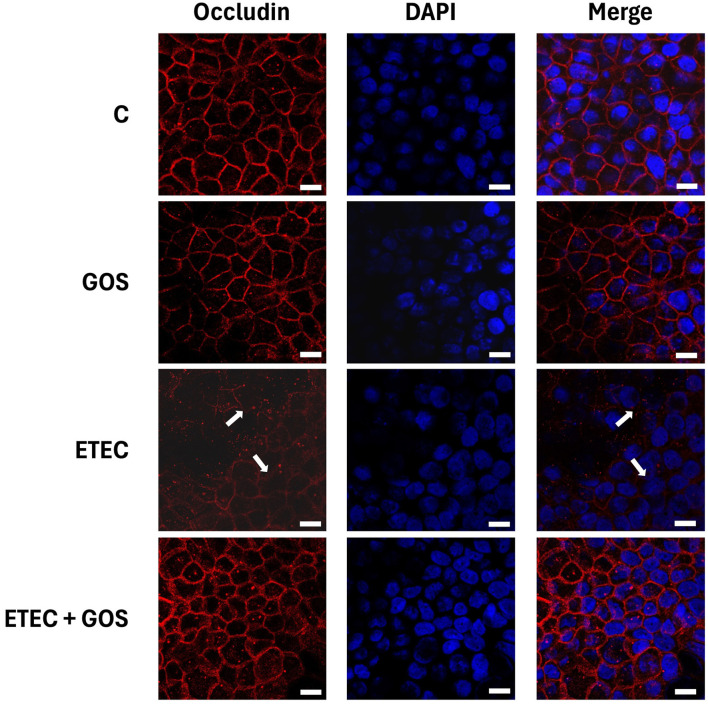
GOS effects on enterotoxigenic *Escherichia coli* (ETEC) F4^+^-induced occludin delocalization in Caco-2 cells, differentiated on permeable filters, assayed by immunofluorescence. Cells were untreated (Control, C), infected with ETEC F4^+^, or treated with 2% GOS, either alone or in combination with ETEC. Cells were then labeled with specific primary antibody for occludin, followed by TRITC-conjugated secondary antibody and 4′,6-diamidino-2-phenylindole (DAPI). In the figure the separate stainings and the merge of them are shown. Interruption of continuous staining of occludin, resulting in dissociation of the protein from membranes in ETEC F4^+^ infected cells is visible (white arrows), as well as regular localization in control cells or treated with GOS either alone or in combination with ETEC F4^+^. Each figure is representative of three independent immunofluorescence assays (40 × magnification). Bars represent 10 μm.

**Figure 7:**
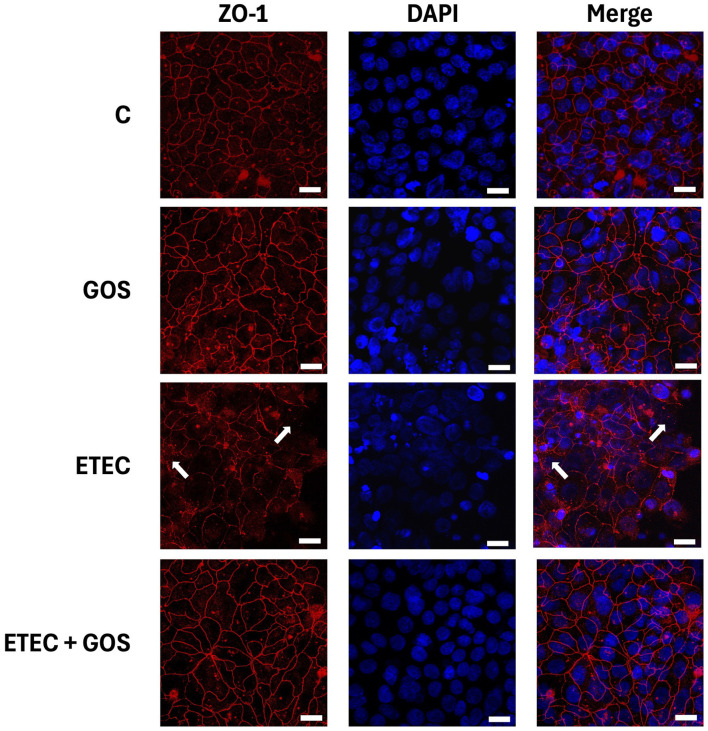
GOS effects on enterotoxigenic *Escherichia coli* (ETEC) F4^+^-induced ZO-1 delocalization in Caco-2 cells, differentiated on permeable filters, assayed by immunofluorescence. Cells were untreated (Control, C), infected with ETEC F4^+^, or treated with 2% GOS, either alone or in combination with ETEC. Cells were then labeled with specific primary antibody for ZO-1, followed by TRITC-conjugated secondary antibody and 4′,6-diamidino-2-phenylindole (DAPI). In the figure the separate stainings and the merge of them are shown. Interruption of continuous staining of ZO-1, resulting in dissociation of the protein from membranes in ETEC F4^+^ infected cells is visible (white arrows), as well as regular localization in control cells or treated with GOS either alone or in combination with ETEC F4^+^. Each figure is representative of three independent immunofluorescence assays (40 × magnification). Bars represent 10 μm.

ETEC F4^+^ has been already shown to damage cell junctions, as previously reported (31). Specifically, TJ proteins of Caco-2 cells are affected by ETEC F4^+^ infection. To evaluate the potential of GOS to protect TJ proteins from pathogen-induced damage, immunolocalization of occludin and ZO-1 was evaluated by immunofluorescence in Caco-2 cells treated with ETEC F4^+^ and GOS, either alone or in combination. As expected, ETEC F4^+^ infection induced occludin and ZO-1 delocalization and disappearance from cell boundaries (Figures 6, 7 for occludin and ZO-1, respectively), indicating cell junction rupture, as also suggested by TEER and phenol red Papp measurements. GOS treatment did not affect TJ proteins, which remained properly distributed on cell boundaries similarly to untreated (control) cells. When Caco-2 cells were treated with GOS in combination with ETEC F4^+^, both occludin and ZO-1 remained correctly localized on cell boundaries, indicating an ability of GOS in preventing cell membrane damage induced by the pathogen.

### 3.5 Modulation of ETEC F4^+^-induced NF-κB activation by GOS

**Figure 8:**
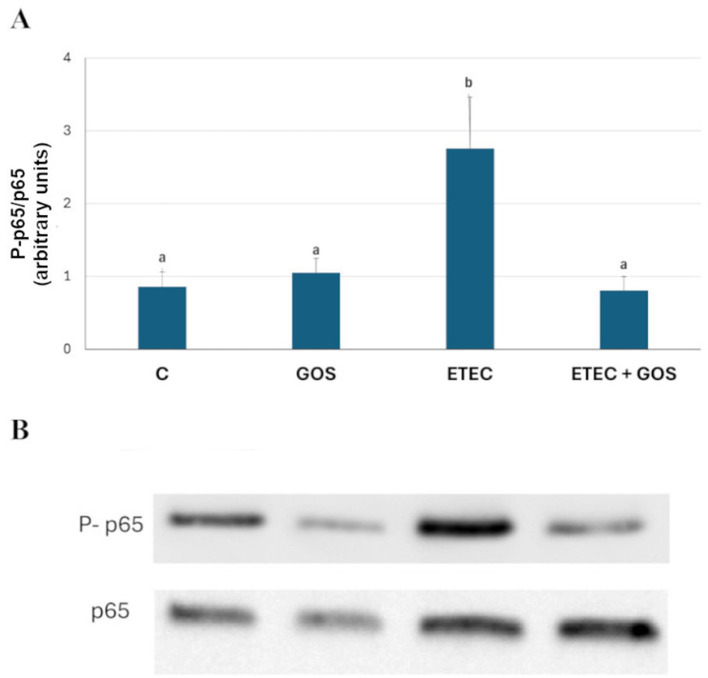
GOS effects on enterotoxigenic *Escherichia coli* (ETEC) F4^+^-induced NF-kB activation in differentiated Caco-2 cells, assayed by Western blotting. Cells were untreated (Control, C), infected with ETEC F4^+^, or treated with 2% GOS, either alone or in combination with ETEC. Cell lysates were fractionated by SDS-PAGE and transferred to nitrocellulose filters. Membranes were incubated with rabbit polyclonal anti-p65 or anti-phosphorylated **(P)**-p65 primary antibodies and then with horseradish peroxidase-conjugated secondary antibodies. **(A)** densitometric values of P-p65 protein, normalized to its corresponding unphosphorylated form, after verifying equal protein loading with α-tubulin, used as internal control. Values represent means ± SD of three independent experiments, carried out in triplicate. Means without a common letter significantly differ (*P* < 0.05). **(B)** representative gel.

**Figure 9:**
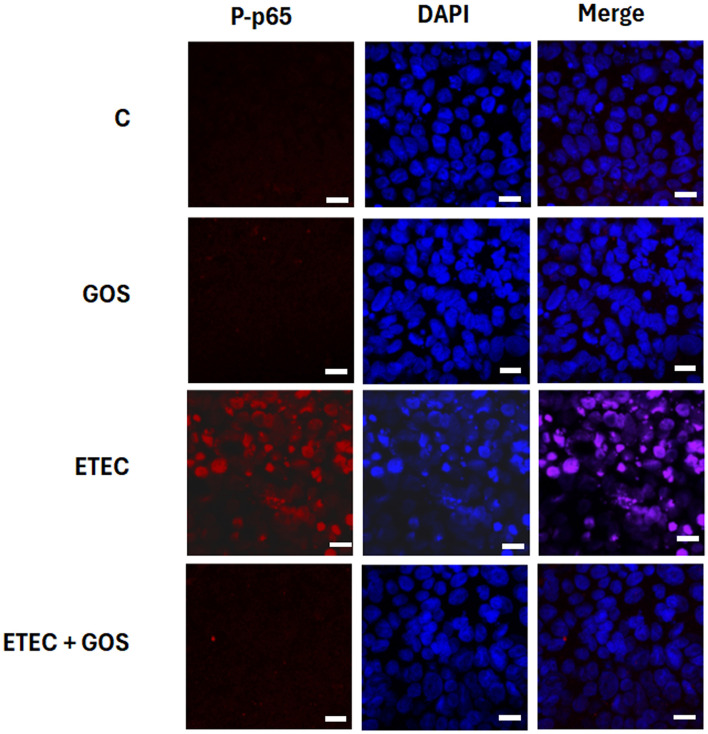
GOS effects on enterotoxigenic *Escherichia coli* (ETEC) F4^+^-induced P-p65 translocation into the nucleus in Caco-2 cells, differentiated on permeable filters, assayed by immunofluorescence. Cells were untreated (Control), infected with ETEC F4^+^, or treated with 2% GOS, either alone or in combination with ETEC. Cells were then labeled with specific primary antibody for P-p65, followed by TRITC-conjugated secondary antibody and 4′,6-diamidino-2-phenylindole (DAPI). In the figure the separate stainings and the merge of them are shown. Each figure is representative of three independent immunofluorescence assays (40 × magnification). Bars represent 10 μm.

In a previous study we showed that ETEC F4^+^ infection induced translocation of phosphorylated (P)-p65 into the nucleus of Caco-2 cells, indicating activation of the NF-κB inflammatory pathway (32). To investigate the ability of GOS to inhibit this inflammatory activity, the phosphorylation status of p65 was assessed by immunofluorescence and Western blot analysis in Caco-2 cells treated with ETEC F4^+^ and GOS, either alone or in combination. The results confirmed that ETEC F4^+^ infection induced a migration of P-p65 into the nucleus, and a significant increase of the phosphorylated form of the p65 subunit (*P* < 0.05), as compared to the phosphorylation level of p65 observed into untreated (control) cells, as shown in Figures 8, 9, respectively. Cell treatment with GOS alone did not induce any change in p65 phosphorylation as compared to control, indicating that GOS did not induce NF-κB activation (Figures 8, 9 for Western blot and immunofluorescence, respectively). In Caco-2 cells treated with ETEC F4^+^ in combination with GOS, phosphorylation level of p65 did not differ from control cells, indicating that GOS were able to prevent the pathogen-induced migration of p65 into the nucleus (Figure 8). The immunolocalization analysis of P-p65 supports the Western blot results, showing that no nuclear localization of P-p65 could be observed in cells treated with ETEC F4^+^ in combination with GOS or treated only with GOS (Figure 9).

### 3.6 Probiotic strains growth promotion by GOS

**Figure 10:**
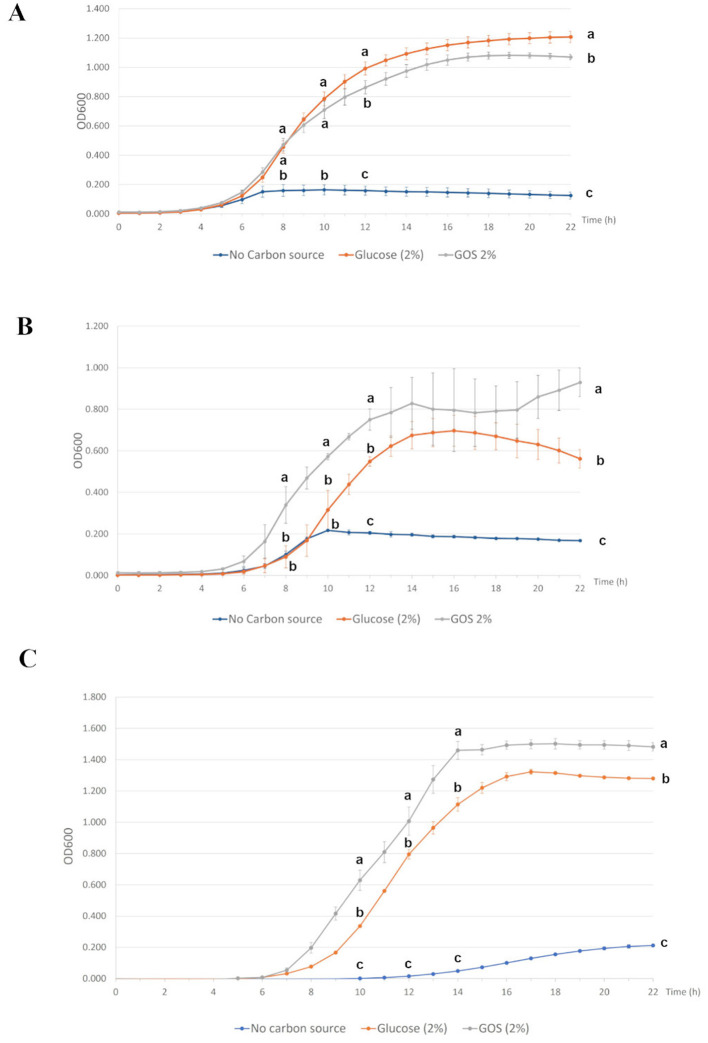
Prebiotic potential of GOS. Growth curves of the three probiotic strains *Lactobacillus amylovorus* ATCC 33198 (LMG9434, **A**), *Lactobacillus amylovorus* DSM 16698 **(B)** and *Limosilactobacillus reuteri* subsp. *porcinus* DSM 110571 **(C)**, grown in MRS without carbon source (negative control), with 2% glucose (positive control) or with 2% GOS. Bacterial growth was monitored by measuring the OD_600_ values at 1 h intervals for 22 h. Values represent means ± SD of one independent experiment, carried out in triplicate. Statistical analysis was performed at different timepoints, chosen as representative of early, mid and late exponential, as well as late stationary growth phases (**A**, **B**: 8, 10, 12 and 22 h; **C**: 10, 12, 14 and 22 h). Within each time point, means without a common letter significantly differ (*P* < 0.051).

The potential prebiotic activity of GOS was assessed by testing its effect on the growth of three probiotic strains of porcine origin. The experimental procedure consisted in replacing the carbon source normally used in bacterial culture medium, namely glucose, with the compound to be tested (i.e. GOS), followed by a comparative evaluation of bacterial growth on the different carbon sources. The growth curves of *L. amylovorus* ATCC 33198, *L. amylovorus* DSM 16698 and *L. reuteri* subsp. *porcinus* DSM 110571 (reported in Figures 10A–C, respectively) clearly showed the ability of all the three probiotic strains to successfully use GOS as a carbon source. Indeed, the respective growth kinetics during 22 h incubation displayed a similar trend between GOS-supplemented and glucose-supplemented conditions, with the typical lag, exponential and stationary phases, differently from the negative control (no carbon source), where no growth at all could be observed (Figures 10A–C). In particular, the overall OD_600_ values of *L. amylovorus* DSM 16698 (8 h, 10 h, 12 h and 22 h time points) and *L. reuteri* subsp. *porcinus* DSM 110571 (10 h, 12 h, 14 h and 22 h time points) resulted significantly higher as compared to glucose-supplemented conditions (*P* < 0.05, Figures 10B, C, respectively). On the other hand, the early and mid exponential phase (8 h and 10 h time points) of the *L. amylovorus* ATCC 33198 growth curve in GOS-supplemented medium overlapped with that in the presence of glucose, although reaching OD_600_ values significantly lower than those observed in the presence of glucose at the late exponential (12 h time point) and at the late stationary (22 h timepoint) phase (*P* < 0.05, Figure 10A).

## 4 Discussion

In recent years, scientific research on prebiotics for animal welfare has expanded significantly. This growing interest can be attributed to several factors, such as enhanced awareness of the fundamental role played by the gut microbiota on health, the greater emphasis on “functional feed additives” designed to improve the overall animal well-being, and the veterinary focus in supporting therapies against infections or gastrointestinal disorders.

Prebiotics are regarded indeed as a promising alternative to antimicrobial growth promoters, whose use has been banned in many countries, including those within the EU, although their application persists in various regions worldwide. This continued usage at sub-clinical doses, has been strongly associated with the emergence and dissemination of antimicrobial resistance, as well as the accumulation of antibiotic residues in animal-derived food products (33).

Within this context, the objective of the present study was to evaluate the potential benefits of GOS in preventing intestinal damage induced by ETEC F4^+^, one of the most predominant causes of post-weaning diarrhea in pigs, still representing an important threat and a major economic loss for the swine industry. For such purpose, the Caco-2 cell line was utilized as differentiated small intestinal enterocytes. Regardless the human origin, Caco-2 cells are widely employed as a universal *in vitro* model of intestinal mucosa barrier, where the AP surface corresponds to the intestinal lumen and the BL side to the underlying basal lamina (23). Caco-2 cells were apically challenged with ETEC F4^+^ to reproduce the pathogen infection occurring in the gut, and GOS protective effects were studied, focusing on microbiota-independent activities.

One of the protective GOS mechanisms involves the inhibition of pathogen adhesion to the intestinal epithelium, that represents the early stage of infection. In our cellular model, GOS significantly reduced adhesion and invasion of ETEC F4^+^, with a biologically relevant reduction of approximately 0.5 log CFU/mL in both assays, corresponding to over 60% inhibition. These findings were consistent with exploratory results obtained from the *ex vivo* model of pig small intestine villi, where GOS inhibited the adhesion of ETEC F4^+^ to porcine villi by more than 50%, suggesting also potential *in vivo* efficacy of GOS. These results are in agreement with previous literature, as Shoaf et al. observed that GOS exhibited an inhibitory effect on both HEp-2 and Caco-2 epithelial cell lines, reducing the adherence of enteropathogenic *Escherichia coli* (EPEC) by 65 and 70%, respectively (34). Similarly, Quintero et al. showed that GOS exerted anti-adhesive effect on *Cronobacter sakazakii* in the same cell lines (35). Searle et al. demonstrated that GOS prevented the adherence and invasion of *Salmonella enterica* serovar Typhimurium both *in vitro* (HT-29-16E cells) and *in vivo* (murine ileal models) (36); while Kittana et al. observed the adhesion reduction of *Citrobacter rodentium* to HEp-2 cells (37). The mechanism by which GOS prevent pathogen adhesion is likely dependent on the structural similarity to the glycan receptors located on the surfaces of host epithelial cells, therefore GOS act as molecular decoys, inhibiting the adherence of pathogens to intestinal epithelial cells by direct competition with fimbrial adhesins. This ability was enhanced in our experimental model by pre-treating ETEC F4^+^ with GOS, before addition to Caco-2 cells, providing mechanistic insights into how GOS may limit early stages of infection (38–40).

When evaluating the potential of GOS to directly impact on ETEC F4^+^ growth, we observed no relevant direct inhibitory effect although pathogen proliferation was modestly yet significantly suppressed at late stationary phase in LB medium added with 2% GOS, as compared to the medium alone. Another study evaluating the GOS antagonistic activity against *E. coli* ATCC 8739 observed a dose-response effect against *E. coli* growth, with a significant reduction starting from 5% GOS (41). Similarly, Mortaz et al. reported that the growth of *Staphylococcus aureus* and *Pseudomonas aeruginosa* was reduced in a concentration-dependent manner when incubated with GOS (42). The mechanisms underlying this direct inhibition of pathogen growth exerted by GOS are still largely unexplored, as a limited number of studies have been performed up to now. In addition, given that LB and DMEM *in vitro* cultures do not fully replicate the gut microbial ecosystems, the modest reduction observed in our study can be potentially indicative of a stronger antimicrobial effect in complex gut environments.

Another beneficial feature of GOS is their positive impact on the intestinal epithelium. In our experimental model, we observed that GOS did not compromise the barrier function of Caco-2 cell monolayers, as both TEER and phenol red Papp remained unchanged for up to 72 h treatment, indicating the maintenance of TJ integrity and suggesting barrier-preserving properties of GOS in physiological conditions. We also observed that GOS alone preserved TJ protein (ZO-1 and occludin) localization at cell boundaries: these findings are supported by the transcriptomic analysis performed by Lafontaine and colleagues in GOS-treated Caco-2 cells, where a modulation of epithelial gene expression relevant to TJ structure in physiological status was observed, indicating that GOS support intestinal epithelial barrier under healthy conditions (43). More interestingly, in our study GOS preserved monolayer integrity from damages induced by ETEC F4^+^, suggesting a potential role as an effective barrier protector against pathogen-induced intestinal disruption. When Caco-2 cell monolayers were challenged with ETEC F4^+^, TEER values dropped and phenol red Papp increased, while in the presence of GOS, these adverse effects were effectively prevented. This observation is consistent with earlier studies showing that GOS could maintain epithelial barrier function and mitigate paracellular leakage under exposure to different stress conditions, cytotoxic such as deoxynivalenol (DON), or inflammatory as dextran-sulfate-sodium salt (DSS) (20, 44). Moreover, occludin and ZO-1 protein localization revealed remarkable protective effects in Caco-2 cells treated with ETEC F4^+^ and GOS. Indeed, while infection with ETEC F4^+^ led to TJ impairment, as shown by delocalization of these key junctional components, GOS treatment was able to prevent the TJ protein delocalization caused by pathogen infection. In a previous study performed in Caco-2 cells, a specific GOS (BimunoGOS) was able to prevent the delocalization of TJ proteins ZO-1 and occludin, as well as adherens junction proteins E-cadherin and β-catenin, induced by DSS treatment (20). Our present data are in agreement with those from Wang and colleagues, who observed that GOS supplementation significantly inhibited the reduction of ZO-1, occludin and claudin-1 protein levels in jejunum, and occludin in ileum of LPS-challenged mice, as well as in IPEC-J2 porcine intestinal cells (45). Based on these data, we can suppose that GOS could be beneficial in preventing gut barrier disruption in challenging conditions, such as pathogen infection. In antibiotic-treated mouse models, GOS induced an increase of TJ protein expression, and also helped to restore eubiosis (46). Moreover, GOS improved barrier function and relieved colonic inflammation in LPS-challenged piglets (47).

Another key finding of the present study was the ability of GOS to inhibit nuclear translocation of p65 following ETEC F4^+^ infection. The absence of p65 activation in GOS-treated infected cells, confirmed by both Western blotting and immunofluorescence assays, indicates that GOS were able to prevent epithelial NF-κB activation. This suggests that GOS may interfere with cell signaling pathways that mediate microbial pattern recognition, such as toll-like receptors, and downstream pro-inflammatory cytokine induction in intestinal cells. Indeed GOS, used both alone or in combination with different stressors (such as DSS, TNF-α or LPS), have been previously shown to exert anti-inflammatory effects on intestinal epithelial monolayers *in vitro* by downregulating the expression and secretion of pro-inflammatory cytokines such as IL-8, IL-1β, and IFN-γ, and by inhibiting the TLR4/NF-κB/TNF-α signaling pathways (20, 48, 49). Tian et al. also observed that supplementing GOS to piglet feed decreased mRNA expression of MyD88-NF-κB signaling in the colonic mucosa of weaned piglets (50).

GOS potential prebiotic activity was also investigated, by analyzing their ability to support the growth of three probiotic strains representative of common inhabitants of piglet gut microbiota, namely *L. amylovorus* ATCC 33198, *L. amylovorus* DSM 16698 and *L. reuteri* subsp. *porcinus* DSM 110571. Indeed, *L. amylovorus* and *L. reuteri* are characteristic bacteria found in piglets, representing the dominant species in the small intestine during and after weaning (51, 52). When used as the sole carbon source in MRS medium, GOS supported the growth of all three probiotic strains, suggesting its potential as prebiotic compound. Among the three strains, *L. amylovorus* DSM 16698 and *L. reuteri* subsp. *porcinus* DSM 110571 displayed enhanced growth performance with respect to the glucose-supplemented condition. Despite its simplified nature compared to *in vivo* or fecal fermentation models, this experimental system offers speed and cost-effectiveness advantages. It enables the acquisition of crucial preliminary information for identifying promising prebiotics, which can then be subjected to more thorough investigation. Another possible application of GOS as prebiotic could be in combination with probiotics, namely in the form of synbiotics. Scientific evidence suggests that supplementing the maternal diet with synbiotics influences the establishment of a balanced offspring's gut microbiota, thereby conferring several health benefits to the piglets (53).

Aside from the role of GOS as promising prebiotic compounds, the novelty of this study lies in demonstrating microbiota-independent effects of GOS against ETEC F4^+^ in an *in vitro* model. These findings, that certainly warrant further investigation, lay the ground for future *in vivo* studies in post-weaning piglets, in which GOS effects against ETEC infection are still largely underexplored. In livestock production, dietary GOS may therefore offer a non-antibiotic based strategy to reduce ETEC colonization, improve gut health, and support performance after weaning.

## 5 Conclusion

Taken together, our findings demonstrate that GOS can enhance gastrointestinal health by exerting multiple protective effects, including competitive inhibition of pathogen adhesion and invasion, preservation of tight junction integrity, prevention of NF-κB nuclear translocation, and growth promotion of beneficial lactobacilli of pig origin, thereby supporting intestinal barrier function through a combination of complementary mechanisms.

Nevertheless, some limitations of the present study should be mentioned. The use of a single epithelial cell line, while informative, does not replicate the complexity of the gut mucosa, which includes also mucus and immune interactions. Moreover, the protective effects were observed at a single GOS concentration, leaving dose-response relationships unexplored. Finally, while the observed findings are consistent with known mechanisms, direct confirmation, such as identifying GOS-fimbriae binding or NF-κB pathway inhibition, remains to be established in further mechanistic and translational studies, that will help to unlock its full therapeutic and commercial potential.

## Data Availability

The raw data supporting the conclusions of this article will be made available by the authors, without undue reservation.
