# Supplementary material for: Protective activity of galacto-oligosaccharides against intestinal damage and inflammation induced by enterotoxigenic Escherichia coli F4+ and evaluation of prebiotic potential
Source: Front Vet Sci. 2026 Feb 2;12:1740099. doi: 10.3389/fvets.2025.1740099 (PMC12908590; doi:10.3389/fvets.2025.1740099)
Supplement: Supplementary file 1 [file Image_1.pdf]

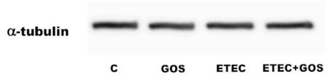


**Figure S1**. GOS effects on enterotoxigenic *Escherichia coli* (ETEC) F4+-induced NF-kB activation in differentiated Caco-2 cells. Cells were untreated (Control, C), infected with ETEC F4+, or treated with 2% GOS, either alone or in combination with ETEC. Cell lysates were fractionated by SDS-PAGE and transferred to nitrocellulose filters. Membranes were incubated with mouse monoclonal anti-α-tubulin primary antibody, used as internal loading control, and then with horseradish peroxidase-conjugated secondary antibody. The Figure shows a representative gel.
