# Supplementary material for: Protective activity of galacto-oligosaccharides against intestinal damage and inflammation induced by enterotoxigenic Escherichia coli F4+ and evaluation of prebiotic potential
Source: Front Vet Sci. 2026 Feb 2;12:1740099. doi: 10.3389/fvets.2025.1740099 (PMC12908590; doi:10.3389/fvets.2025.1740099)
Supplement: Supplementary file 2 [file Image_2.pdf]

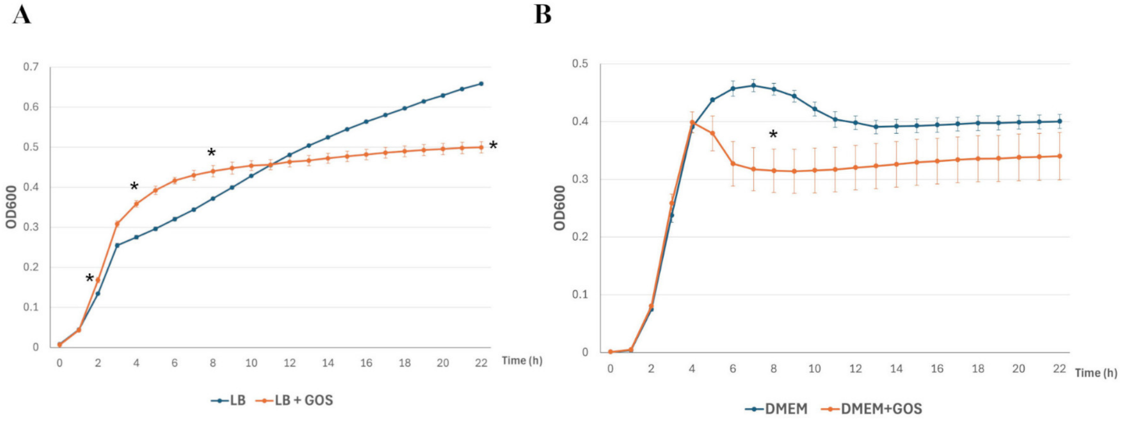


**Figure S2**. GOS effects on enterotoxigenic *Escherichia coli* (ETEC) F4+ growth in LB bacterial medium (panel A) or DMEM Caco-2 cell medium (panel B). ETEC F4+ was grown either alone (LB, DMEM) or in the presence of 2% GOS (LB + GOS, DMEM + GOS). Bacterial growth was monitored by measuring the OD600 values at 1 h intervals for 22 h. Values represent means ± SD of one independent experiment, carried out in triplicate. Statistical analysis was performed by Student's t-test at different timepoints, chosen as representative of early, mid and late exponential, as well as late stationary growth phases (2, 4, 8 and 22 h, respectively, $^*P < 0.01$ within each time point).
